# Familial human prion diseases associated with prion protein mutations Y226X and G131V are transmissible to transgenic mice expressing human prion protein

**DOI:** 10.1186/s40478-018-0516-2

**Published:** 2018-02-20

**Authors:** Brent Race, Katie Williams, Andrew G. Hughson, Casper Jansen, Piero Parchi, Annemieke J. M. Rozemuller, Bruce Chesebro

**Affiliations:** 1 Laboratory of Persistent Viral Diseases, Rocky Mountain Laboratories, National Institute of Allergy and Infectious Diseases, National Institutes of Health, 903 South Fourth Street, Hamilton, MT 59840 USA; 2 Laboratorium Pathologie Oost Nederland, Hengelo, The Netherlands; 3 IRCCS Institute of Neurological Sciences, Bologna, Italy; 4 Department of Experimental, Diagnostic and Specialty Medicine, University of Bologna, Bologna, Italy; 5 Department of Pathology, VU University Medical Center, de Boelelaan 1117, 1081 HV Amsterdam, The Netherlands; 6 Department of Pathology, University Medical Center Utrecht, Utrecht, The Netherlands

## Abstract

Human familial prion diseases are associated with mutations at 34 different prion protein (PrP) amino acid residues. However, it is unclear whether infectious prions are found in all cases. Mutant PrP itself may be neurotoxic, or alternatively, PrP mutation might predispose to spontaneous formation of infectious PrP isoforms. Previous reports demonstrated transmission to animal models by human brain tissue expressing 7 different PrP mutations, but 3 other mutations were not transmissible. In the present work, we tested transmission using brain homogenates from patients expressing 3 untested PrP mutants: G131V, Y226X, and Q227X. Human brain homogenates were injected intracerebrally into tg66 transgenic mice overexpressing human PrP. Mice were followed for nearly 800 days.

From 593 to 762 dpi, 4 of 8 mice injected with Y226X brain had PrPSc detectable in brain by immunostaining, immunoblot, and PrP amyloid seeding activity assayed by RT-QuIC. From 531 to 784 dpi, 11 of 11 G131V-injected mice had PrPSc deposition in brain, but none were positive by immunoblot or RT-QuIC assay. In contrast, from 529 to 798 dpi, no tg66 mice injected with Q227X brain had PrPSc or PrP amyloid seeding activity detectable by these methods. Y226X is the only one of 4 known PrP truncations associated with familial disease which has been shown to be transmissible. This transmission of prion infectivity from a patient expressing truncated human PrP may have implications for the spread and possible transmission of other aggregated truncated proteins in prion-like diseases such as Alzheimer’s disease, Parkinson’s disease and tauopathies.

## Introduction

Prion diseases are rare fatal neurodegenerative diseases of humans and animals which are transmissible by exposure to diseased tissues via ingestion, injection or transplantation. These diseases are often characterized by spongiform degeneration or vacuolation of gray matter, astrogliosis and microgliosis, and deposition of a partially proteinase K-resistant disease-associated form of the normal host prion protein (PrP) [5, 24]. The disease-related PrP, known as PrP scrapie (PrPSc), is generated by a seeded conversion mechanism where small aggregates of PrPSc bind normal PrP and mediate its conversion to PrPSc [6].

A similar prion-like seeded polymerization mechanism appears to be responsible for the formation of protein aggregates involving α-synuclein, Aβ and tau in Parkinson’s disease, Alzheimer’s disease, and tauopathies [13, 48]. These findings have increased interest in prion diseases, and there is hope that there might be a crossover in potential therapies for prion diseases and prion-like diseases.

In humans, prion diseases can be divided into several categories based on presumed etiologies [5, 11]: sporadic Creutzfeldt-Jakob disease (CJD), iatrogenic CJD associated with injection or grafting of infected tissue (growth hormone, dura and cornea), variant CJD associated with exposure to bovine spongiform encephalopathy (BSE)-contaminated beef, and genetic/familial prion disease associated with inherited PrP mutations.

To date, mutations at 34 different sites in the human prion protein gene are associated with development of genetic prion diseases in an autosomal dominant pattern with heterogeneous phenotypes [27]. However, genetic prion diseases do not always fit precisely within the classical definition of prion disease, i.e. rapid clinical decline, spongiform degeneration, gliosis and presence of partially protease-resistant PrP. In contrast, genetic prion diseases usually display prolonged clinical course, variable spongiform degeneration, variation in the molecular size of PrP detected in disease-associated deposits, and presence of abnormal PrP in an amyloid form. Genetic prion diseases can be subdivided into different groups based on clinical/pathological characteristics. These include genetic/familial CJD, Gerstmann-Sträussler-Scheinker disease (GSS) and fatal familial insomnia (FFI). GSS disease is unusual in that PrPSc is mostly in the amyloid form which is deposited either as multifocal amyloid plaques in the neuropil or as perivascular plaques consistent with cerebral amyloid angiopathy (CAA) [17]. In GSS and PrP-CAA, immunoblotting reveals proteinase K (PK)-resistant PrP bands approximately 7–8 kD in size which correlate with the presence of amyloid PrPSc and are distinct from the larger bands usually seen in genetic CJD or FFI [31, 33, 42].

Human familial prion diseases have been extensively studied by modeling in mice expressing transgenes which express a human PrP mutation superimposed on a normal PrP sequence. PrP from several species including mouse, human, hamster, cow and bank vole have been generated. These models have recently been compared in an elegant review [50]. Overall the results indicate that many of these models develop spontaneous disease with similarities to their human disease counterparts. Transmissible prion infectivity has been found in some, but not all, of these models [1, 12, 21, 49], suggesting that presence of prion infectivity is not absolutely required for the development of these signs of clinical neurological disease. One hypothesis suggests that PrP mutations linked to familial prion diseases can generate infectious prions in some afflicted patients [19]; however, an alternative possibility is that in other patients mutant PrP molecules might induce neurodegeneration by disruption of normal CNS functions without production of infectious prions [28, 50].

Detection of prion infectivity in prion disease-affected human brain has also been studied by injection of human patient brain into susceptible animals. Transmission of sporadic, iatrogenic and variant forms of CJD has been demonstrated previously [3, 4, 16, 18, 25, 44, 47]. However, results of transmission experiments using familial prion disease brain have been more variable. Transmission experiments using human CNS tissue of familial prion disease patients have been done with ten of the 34 PrP mutations known to be associated with prion disease. In these experiments, seven mutations gave positive transmissions to rodents or primates. These included GSS-associated mutations (P102L, A117V, F198S) [3, 34, 44], FFI mutation (D178N with 129 MM) [43] and familial CJD mutations (D178N with 129VV, E200K, V210I, M232R) [3, 26, 44]. In contrast, no transmission was reported for mutations P105L, Y145X, or Y163X [27, 44]. It remains unclear whether these negative cases lacked any prion infectivity or were the result of low infectivity levels in the brain regions analyzed or the use of less sensitive host animals for the transmissions. Interestingly, two of these negative transmission cases involved patients with a mutation which created a stop codon resulting in PrP truncation (Y145X and Y163X), suggesting that PrP truncations might not generate spontaneous prion infectivity in vivo in humans.

In the present study, we performed transmission experiments using brain tissue of human patients expressing three previously untested PrP mutations (Y226X, Q227X, and G131V) [22, 23, 30]. Tg66 transgenic mice expressing human PrP at a level 8–16-fold higher than physiological levels [37, 38] were used as recipients. Interestingly, two of these human patients had PrP mutations resulting in a stop codon at positions 226 or 227, thus lacking the final 5–6 amino acid residues of PrP as well as the C-terminal glycophosphatidylinositol (GPI) anchor group. Thus, the mutant PrP in these two patients was remarkably similar to the anchorless PrP expressed in tg44 transgenic mice [8, 10], which showed severe cerebral amyloid angiopathy (CAA) after scrapie infection, similar to the Y226X patient. In this work, we observed transmission to tg66 mice by tissue from the Y226X patient starting at 593 dpi, and possible transmission of G131V starting at 531 dpi. In contrast, no transmission by Q227X was seen by 798 dpi which was the latest time-point analyzed. Y226X is the first human PrP mutant associated with PrP truncation which has been found to be transmissible.

## Methods

### Human donor tissue

Tissues from all three patients with mutations in the human PrP gene (*PRNP*) were obtained from Dr. Annemieke Rozemuller at the Dutch Surveillance Center for Prion Diseases, University Medical Center Utrecht (UMCU), Utrecht, The Netherlands and the VU University Medical Center in Amsterdam, The Netherlands. Frozen brain tissue from the 55-year old Y226X mutant patient (UMCU #S08–005) [23] was not available. Therefore, brain tissue from the cingulate gyrus was provided as formalin-fixed (three days), paraffin-embedded tissue sectioned and dried on glass slides. To create the brain homogenate used for inoculation into mice, tissue from 10 slides (approximately 1 cm^2^ each) was de-paraffinized and rehydrated using standard protocols, and then scraped from the slides using a razor blade. The resulting material was minced into small pieces (less than 0.5 mm^2^) with a scalpel and added to a total volume of 500 μl phosphate buffered saline (PBS). This suspension was vortexed and sonicated for several rounds of 30 s each until no large pieces could be observed.

Tissues from a 42-year old patient with the PRNP Q227X mutation (UMCU #S07–176) [23] and from a 52- year old patient with the PRNP G131V mutation (UMCU #S04–346) [22] were provided as frozen brain samples from the middle frontal gyrus of the frontal lobe. The frozen tissues were thawed at the Rocky Mountain Laboratory (RML) and prepared as 20% *w*/*v* homogenates in PBS using an OMNI tissue homogenizer. Prior to inoculation, 20% brain homogenate aliquots were thawed, sonicated and further diluted to 10% or 1% brain homogenate in PBS for stereotactic microinjection (1ul) or macroinjection (30ul) respectively.

As shown in the original references [22, 23], the PRNP codon 129 genotype for all three patients was 129MV, and all three patients expressed one mutant PrP allele and one nonmutant allele. In the cases of Y226X and Q227X the mutant PrP was associated with 129V, whereas in the case of G131V the mutant PrP was associated with 129M. Brain tissue of all three patients had PrPSc detectable by IHC using antibody 3F4, and the Q227X and G131V patients also had protease-resistant PrP detectable by immunoblotting. Unfortunately, the Y226X patient brain was only available as formalin-fixed tissue and no immunoblotting was possible.

### Mice

All mice were housed at RML in an AAALAC-accredited facility in compliance with guidelines provided by the Guide for the Care and Use of Laboratory Animals (Institute for Laboratory Animal Research Council). Experimentation followed RML Animal Care and Use Committee approved protocol #2014–095. Generation of tg66 transgenic mice expressing human PrP were described previously [37]. These mice are on a FVB/N genetic background, and are homozygous for a transgene that encodes human prion protein MM129. Tg66 mice overexpress human PrP at 8–16-fold levels higher than normal physiologic levels and have been shown to be susceptible to vCJD, sCJD and mouse-adapted 22 L scrapie [37, 38].

### Intracerebral injections of mice with human brain homogenates

Young adult (6–8 week) tg66 mice were infected with brain homogenates derived from three human donors with different PRNP mutations (Y226X, Q227X, G131V). Each mouse was anesthetized with isoflurane and then intracerebrally injected in the left hemisphere with 30 μl of a 1% brain homogenate stock diluted in PBS. Alternatively, some mice were stereotactically microinjected in the striatum with 1 μl of 10% brain homogenate. This method was used to facilitate possible early detection of PrPSc replication at the site of the needle track, as was demonstrated in previous experiments at 3 to 7 days post-microinjection of mouse scrapie [9].

The details of the microinjection were as follows: Mice were anesthetized with isoflurane and prepared for aseptic surgery by shaving the dorsal surface of the skull and applying a chlorhexidine-based surgical scrub. Ophthalmic ointment was applied to each eye and the mouse was then transferred to and positioned on a stereotaxic frame (David-Kopf Instruments, Tujunga, CA). A 1-cm midline incision was made in the skin over the dorsal surface of the skull, and the skull was exposed to allow the positioning of a drill over the Bregma point of reference. From Bregma, the coordinates used were + 1 mm anteroposterior, + 1.7 mm lateral, and − 3 mm ventral to the skull surface. These coordinates were selected to target the center of the left striatum and avoid passing through any ventricle. 10% brain homogenates were injected with Nanofil syringes (World Precision Instruments, Sarasota, FL) and steel bevel needles (33-gauge diameter for Q226X and G131V and 26-gauge diameter for the Y226X brain homogenate) into the striatum at a rate of 0.5 μl/min with a total of 1.0 μl per mouse controlled with a pump (UltraMicroPump III with a Micro4 pump controller; World Precision Instruments). The needle was kept in place for 2 min following injection to avoid any reflux of the brain homogenate (BH) solution. The skin incision was closed with sutures. The patency of the needles was verified prior to and after injections. Following surgery mice were placed on a heating pad until fully recovered.

### Clinical observations

Following inoculation, mice were monitored for onset of prion disease signs by experienced observers. Observation of onset and progression of the subtle clinical disease in these experiments was very difficult, as the extremely long incubation periods overlapped with the natural lifespan of the mice. In many cases the slowly progressive neurologic signs were complicated with other age-related concurrent conditions. Mice were euthanized when they displayed consistent neurologic signs including tremors, head bobbing, weakness, ataxia, circling or kyphosis or when other non-neurologic conditions such as weight loss or neoplasia necessitated euthanasia. Following euthanasia brains were removed, and half of the brain was placed into formalin and half of the brain was frozen for biochemical analysis.

### Immunoblotting

Initially, brain samples from tg66 mice were screened for proteinase K (PK)-resistant PrP (PrPres) by immunoblot as previously described [38]. No bands were visualized using this technique so additional testing was performed using an adapted sodium PTA procedure [45] for each sample. For each experimental group, 8–9 mice were analyzed for PrPres following sodium phosphotungstic acid (PTA) precipitation. For each mouse, 20% *w*/*v* brain homogenates (BH) were made in phosphate buffered saline (PBS) using a mini-bead beater system set to homogenize for 45 s, and were stored frozen at − 20 C. For further use, homogenates were thawed and diluted in PBS to create 10% homogenates. 500 μl of a 10% BH was mixed with an equal volume of 4% Sarkosyl, vortexed, and incubated in a water bath at 37 °C for 30 min. Benzonase (5 U/μl) and magnesium chloride (0.2 M) were then added to final concentrations of 25 U/ml and 0.001 M, respectively. Samples were vortexed and incubated in a water bath at 37 °C for 45 min. Centrifugation at 5000×*g* for 5 min at room temperature was performed, and the supernatant was transferred to a new tube. PK was added to a final concentration of 20 μg/ml, and the mixture was vortexed and incubated in a water bath at 37 °C for 1 h. The reaction was stopped with a 5 mM final concentration of Pefabloc. Four percent sodium PTA and 34 mM magnesium chloride, pH 7.4, were added to final concentrations of 0.3% and 2.73 mM, respectively, and the solution was incubated in a water bath at 37 °C for 1 h. Samples were then centrifuged at 16,000×*g* for 30 min at 37 °C, and the supernatants were discarded. Pellets were then resuspended in 200 μl of PBS-EDTA (40 ml of 0.5 M EDTA and 60 ml of PBS, pH 7.4), incubated for 30 min in a 37 °C water bath, and then centrifuged at 16,000×*g* for 30 min at 37 °C. The supernatants were again discarded, and the pellet was resuspended in 60 μl of Laemmli sample buffer, vortexed, and boiled for 5 min. 20 μl was loaded into a single lane on a 16% Tris-glycine gel (Invitrogen, Thermo Fischer Scientific) and electrophoresed. Gels were transferred to polyvinylidene difluoride membranes with the iBlot transfer system using a 7-min transfer, program 3 (Life Technologies). Membranes were probed with a 1:3000 dilution of mouse anti-PrP antibody 3F4. The secondary antibody was peroxidase-conjugated rabbit anti-mouse IgG at 1:80,000 (Sigma), and immunoreactive bands were visualized with film using a SuperSignal West Femto (Thermo Scientific) detection system. A panel of molecular weight marker proteins was run on each gel (Bio-Rad, Kaleidoscope or SDS Low Range), and the approximate size of each PrP band was calculated by extrapolation using these markers.

### Immunohistochemistry and histology

Brains were removed, cut in half in the sagittal plane, and one half of each brain was placed in 10% neutral buffered formalin for 3 to 5 days. Tissues were then processed by dehydration and embedding in paraffin. Sections were cut using a standard Leica microtome, placed on positively charged glass slides, and air-dried overnight at room temperature. On the following day slides were heated in an oven at 60 °C for 20 min. Neuropathology was assessed on hematoxylin and eosin (H&E) stained sections. H&E staining was performed according to the manufacturer’s (Shandon) instructions; hematoxylin incubation of 12 min, eosin incubation of 4 min.

For prion protein detection, deparaffinization and hydration of tissue sections was performed manually using Pro-Par solvent and graded alcohols to distilled water. Antigen retrieval was accomplished using a Biocare Medical DC2002 Decloaking Chamber and Citrate Buffer pH 6.0 (0.01 M), ~ 20 min at 120 °C and 20 PSI. For staining of prion protein, a biotinylated monoclonal anti-prion antibody 3F4 (Covance Research Products) was used at a 1:50 dilution in antibody dilution buffer (Ventana ADB250), and applied for 60 min at 37 °C followed by detection using a DABMap detection kit and hematoxylin counterstain. Staining was performed on the automated Discovery XT staining system (Ventana Medical Systems).

PrPSc was defined on 3F4-stained immunohistochemistry slides of brain tissue as fine, coarse or plaque-like deposits of brown-stained material which was seen in tg66 mice injected with brain from familial prion disease patients with PrP mutations, but which was not seen in aged-matched uninjected control tg66 mice. Furthermore, by these criteria, PrPSc was not detected by immunohistochemistry (IHC) in tg66 mice injected with Q227X human brain (see results). Therefore, Q227X-injected mice also served as age-matched negative controls.

For detection of microglia, polyclonal rabbit anti-Iba1 antiserum was used. This antiserum was generated by immunization of rabbits with a 14 amino acid peptide from the C-terminus of the Iba1 protein as previously described [20], and was a generous gift from Dr. John Portis. For detection of astroglia, a polyclonal rabbit anti-GFAP antibody (DAKO Cytomation) was used. The Discovery XT staining system (Ventana Medical Systems) was used for deparaffinization, antigen retrieval and staining using a RedMap detection kit and hematoxylin counterstain. For Iba1, antigen retrieval was done using the standard CC1 protocol (cell conditioning buffer containing Tris-Borate-EDTA, pH 8.0, ~ 44 min at 100 °C). Anti-Iba1 was used at a 1:2000 dilution and applied for 40 min at 37 °C. The secondary antibody was biotinylated goat anti-rabbit IgG (Biogenex Ready-to-use Super Sensitive Rabbit Link) and was applied for 40 min @ 37 °C. For GFAP staining antigen retrieval was done using the mild CC1 protocol (cell conditioning buffer containing Tris-Borate-EDTA, pH 8.0, ~ 12 min at 100 °C). The anti-GFAP antibody was used at a dilution of 1:3500 in antibody dilution buffer, applied for 16 min at 37 °C. The secondary antibody was biotinylated goat anti-rabbit IgG described above and was applied for 16 min at 37 °C.

Sections stained with H&E, 3F4, Iba1 and GFAP were scanned with an Aperio ScanScope XT (Aperio Technologies, Inc.) and analyzed and photographed using Aperio Imagescope software.

Thioflavin S staining was performed to determine the amyloid nature of the plaques and aggregates observed in the transgenic mouse brains. Thioflavin S (SIGMA, practical grade) was applied as a 1% wt./vol. solution in distilled water for 5 min at room temperature with no light. Slides were examined within 24 h post-staining, and photomicrographs were taken using an Olympus BX51 microscope and Microsuite FIVE software.

### RT-QuIC

RT-QuIC reactions were performed as previously described using recombinant bank vole PrPsen as substrate (residues 23 to 230; Methionine at residue 109; accession no. AF367624) [29]. PrP from bank voles has proven to be a very good at detecting PrP amyloid seeding activity by RT-QuIC assay in a wide variety of species [29]. Briefly, sample brains were homogenized to 10% (*w*/*v*) in PBS. Homogenate supernatants were then collected following a 2 min clearance step at 2000 x g. Samples were then 10-fold serially diluted in 0.05% SDS (sodium dodecyl sulfate, Sigma)/PBS/N2 (Gibco) to yield 10^− 3^ brain tissue concentrations. Four independent wells were tested for each mouse brain sample. 2 μl sample volumes were added to reaction wells of a black 96-well, clear bottom plate (Nunc) containing 98 μl of RT-QuIC reaction mix, resulting in final concentrations of 0.001% SDS, 10 mM phosphate buffer (pH 7.4), 300 mM NaCl, 0.1 mg/ml rPrPSen, 10 μM thioflavin T (ThT), 1 mM ethylenediaminetetraacetic acid tetrasodium salt (EDTA). The plate was then sealed with a plate sealer film (Nunc) and incubated at 42 °C in a BMG FLUOstar Omega plate reader with a repeating protocol of 1 min shaking (700 rpm double orbital) and 1 min rest throughout the indicated incubation time. ThT fluorescence measurements (450 +/− 10 nm excitation and 480 +/− 10 nm emission; bottom read) were taken every 45 min. Data was normalized to the percentage of maximum fluorescence obtained from the strongest signal in the run.

Each mouse was tested on a minimum of two independent runs. Results were similar for each sample between the separate experiments. To distinguish RT-QuIC positive and negative results, we established criteria based on negative control results and titrations of positive controls. To be scored positive, a sample had to show greater than 50% of maximal seeding activity in 2 or more out of 4 wells prior to 30 h of reaction time.

### Second passage transmission experiments

For each PrP mutant human brain sample, three first passage tg66 mice were selected for second passage into tg66 mice. Tg66 mouse brains were homogenized to a 20% homogenate as described in the immunoblotting section. For injection, the 20% brain homogenates were further diluted to a 1% homogenate in a phosphate buffered balanced salt solution with 2% fetal bovine serum. Each recipient mouse was anesthetized with isoflurane and intracerebrally injected with 30 μL of 1% homogenate. Nine to 12 recipient mice were injected per donor mouse. Following inoculation, mice are being monitored for onset of prion disease signs as described above.

## Results

### Transmission experiments using brain tissue from a patient expressing a Y226X PrP mutation

Transmission of prion infection was attempted using brain tissue from a human patient with familial prion disease who was heterozygous for a mutant PRNP allele (Y226X) resulting in expression of truncated PrP protein [23]. This patient died at age 57 after a 27-month course with severe progressive memory and visuospatial dysfunction followed by akinesis and mutism. By microscopic examination of the brain severe cerebral amyloid angiopathy (CAA) was seen in numerous areas, and amyloid was intensely stained by anti-PrP antibodies. Small focal tau accumulations were also noted but neurofibrillary tangles were absent.

Transmission experiments were carried out by intracerebral injection of patient brain homogenate into tg66 transgenic mice expressing human PrP. Homogenates were made from three-day formalin-fixed paraffin-embedded sections as described in the methods. At various times starting at 77dpi, injected mice were euthanized for examination of brain tissue by IHC. Mice studied at 77, 240 and 509 dpi were negative for PrPSc by IHC and showed no clinical signs suggestive of neurological disease (not shown). However, from 593 to 762 dpi, 4 of 8 mice tested had PrPSc detectable both by IHC using monoclonal antibody 3F4 and immunoblot by using the phosphotungstic acid (PTA) enrichment method with detection by 3F4 (Table 1).

**Table 1:** Transmission study of human, genetic mutant Y226X PrP isolate to tg66 transgenic mice expressing human PrP

Mouse number	DPI	PrPSc IHC	PrPSc western blot	Clinical TSE suspect	Clinical notes (reason for euth) & relevant necropsy findings
B321–3	77^a^	–	nt	No	normal
B325–2	77^a^	–	nt	No	normal
B326–1	240^a^	–	nt	No	normal
B326–2	240^a^	–	nt	No	normal
B326–3	509^a^	–	nt	No	normal
B326–4	509^a^	–	nt	No	normal
B323–1	593	+	+	No	injury necessitating euthanasia
B322–1	601	+	+	Yes	urine scalding, ataxic, poor nest, thin, dilated, thickened uterus, consolidated lung lobe
B322–2	609	–	–	Yes	thin, circling, kyphosis, poor nesting, ataxic, tippy-toed gait
B323–2	680	–	–	Yes	thin, hunched, progressive paraparesis, bilaterally distended and inflamed uterus
B324–1	716	+	+	No	tremor, mild ataxia, abnormal respirations, good body condition
B323–3	718	+	+	Yes	Weight loss, poor coat quality, hunched posture
B326–5	720^a^	–	–	No	old age, thin
B324–2	762	–	–	Yes	thin, hunched, wobbly

^a^- Indicates mice that were stereotactically microinjected into the striatum. As described in the methods, this technique was used to facilitate possible early detection of PrPSc replication at the site of the needle track, as was previously demonstrated [9]. Mice injected using this technique were euthanized electively and tissues were processed to directly screen the injection needle track and adjacent brain by IHC for any PrP replication. Mice without asterisks were intracerebrally inoculated with a 30ul volume of brain homogenate and euthanized when they developed neurologic signs consistent with prion infection or when they developed conditions requiring euthanasia for humane reasons. nt = not tested

**Fig. 1:**
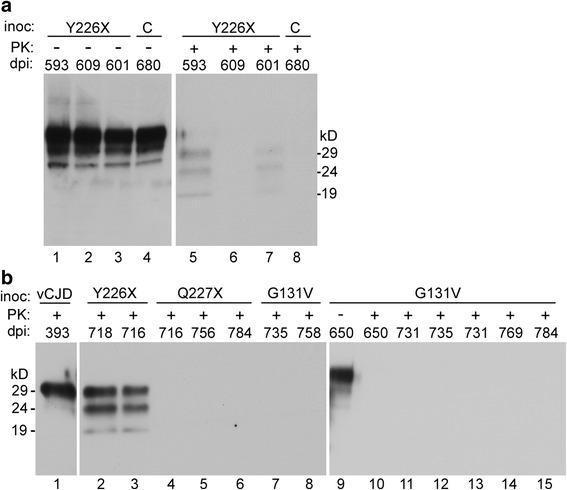
Immunoblot detection of PrP in brain tissues from tg66 mice. Mice were intracerebrally injected with brain homogenates from human GSS patients expressing PrP mutants Y226X, Q227X and G131V. Mice are identified by their days post-injection (dpi), and can be cross-checked by this identifier in the Tables. Prior to analysis, all samples were concentrated using a PTA procedure as described in the methods, except for panel **b**, lane 1 (vCJD), which was processed by the standard protocol [38]. Panel **a** shows one control uninfected mouse (C) and three tg66 mice infected with brain from the patient with mutant Y226X. Samples in lanes 1–4 had no PK treatment (−), and samples in lanes 5–8 were treated with PK at a concentration of 20 μg/ml (+). Following PK digestion, two Y226X injected mice (593 and 601) showed faint PK resistant PrPSc bands at 29, 24 and 19kD (lanes 5 and 7) as indicated on right margin. Samples in lanes 1–4, which were not PK-treated, showed similar PrP bands in all the mice. Since this PrP pattern was also found in the uninfected control, this would appear to be PTA-precipitated aggregated PK-sensitive PrPC, which was present in all tg66 mice. In panel b, two additional Y226X injected mice (718 and 716) in lanes 2 & 3 had a strong PrPSc signal. In addition, three Q227X injected mice (lanes 4–6) and eight G131V- injected mice (lanes 7–15) had no detectable PrPSc. Mouse 650 was analyzed both without and with PK treatment in lanes 9 and 10. The blot in panel a was probed with a combination of monoclonal anti-PrP antibodies 3F4 and SAF32, and panel **b** was probed with antibody 3F4 alone. Approximate molecule weights calculated for each PK-resistant band are shown in margins

In immunoblots, mice euthanized at 593 and 601 dpi showed weak PrPSc bands at 29, 24 and 19 kD, whereas a mouse euthanized at 609 dpi had no bands detectable (Fig. 1a). In a separate blot (Fig. 1b), mice euthanized at 716 and 718 dpi showed strong PrPSc bands at the same sizes as were seen in Fig. 1a, but mice euthanized at 680 and 762 dpi had no bands visible (not shown). No bands were detected in experiments where PTA precipitation for PrPSc enrichment was not used.

**Fig. 2:**
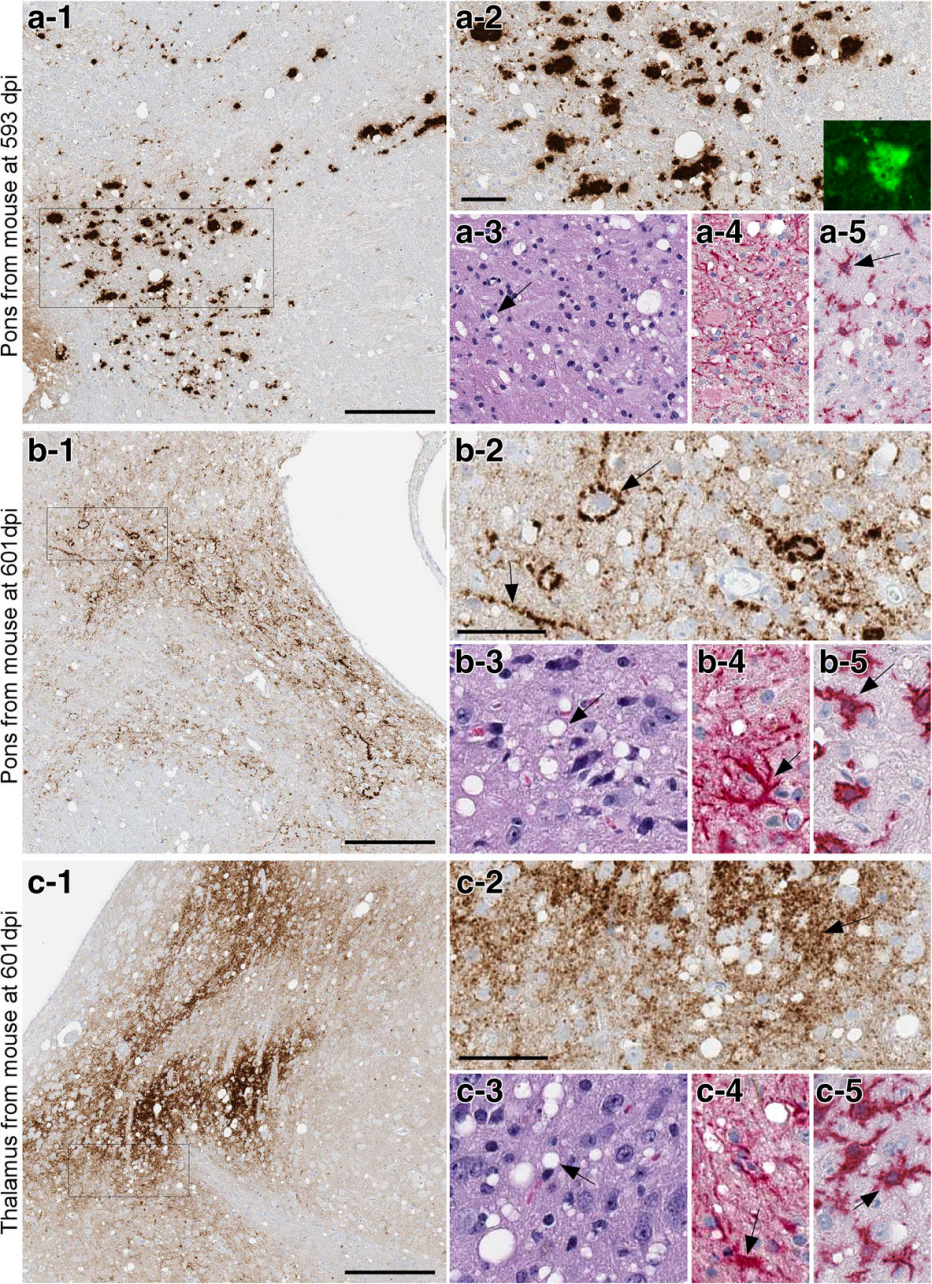
Immunohistochemistry and neuropathology of tg66 mice injected with Y226X human brain homogenate. Panel **a** Pons region of a mouse euthanized at 593 dpi. PrPSc was detected by IHC using biotinylated antibody 3F4 as described in the methods (panel a-1). Large and medium-sized PrPSc deposits are seen at higher magnification (a-2). Inset in a-2 shows Thioflavin S staining of one aggregate. Typical prion disease vacuolation (arrow) is shown by H&E staining (a-3), and astrogliosis and microgliosis (arrow) are shown by anti-GFAP staining (a-4) and anti-Iba1 staining (a-5). Panel **b** Pons region of a mouse euthanized at 601 dpi. PrPSc staining showed smaller coarse deposits (b-1), and perineuronal and linear axonal staining (arrows) could be seen at higher magnification (b-2). Vacuolation, astrogliosis and microgliosis (arrows) was also prominent in this same area (b-3, b-4, b-5). Panel **c**: Thalamus of same mouse shown in panel **b** showed slightly finer staining of PrPSc at both low (c-1) and high (c-2) magnification. Prominent vacuolation (c-3), astrogliosis (c-4) and microgliosis (c-5) was also noted (arrows). Scale bars shown in a-1, b-1 and c-1 are 200 μm, scale bars shown in a-2, b-2 and c-2 are 50 μm and apply to each subsequent panel within the same figure letter

In IHC experiments, the mouse euthanized at 593 dpi had plaque-like PrPSc deposits in pons (Fig. 2a), and some of these deposits stained with Thioflavin S, indicating that they had amyloid properties. In the mouse euthanized at 601 dpi, perineuronal deposits were seen in pons (Fig. 2b) and coarse neuropil deposits were found in the thalamus (Fig. 2c). Gray matter vacuolation, astrogliosis and microgliosis, all typical of prion disease were also noted in the regions of PrPSc deposition (Fig. 2). Two additional mice euthanized at 716 and 718 dpi also had PrPSc deposits in most of these same brain regions (Table 2). Thus, detection of PrPSc by IHC correlated exactly with detection by immunoblotting.

**Table 2:** Characterization and location of PrPSc deposits in tg66 mice injected with Y226X human brain tissue

	Brain regions^a^				
Dpi^b^	Pons	Thalamus	Superior Colliculus	Hypothalamus	Cerebral cortex
593	P	C	C	P	neg
601	P&PN	C	C	PN	PN
716	P&PN	neg	neg	P	P (rare)
718	P&PN	neg	C (rare)	neg	C (weak)

^a^-Types of PrPSc deposits seen in the indicated brain regions: P = plaque-like, PN = perineuronal linear or pericellular deposits, C = coarse deposits in neuropil, neg = negative

^b^-Days post-injection with human Y226X brain tissue

Various clinical signs were also noted in 5 of the 8 mice euthanized between 593 and 762 dpi (Table 1). However, because of the poor correlation between clinical signs and presence of PrPSc, these results did not allow a definitive conclusion as to whether these signs were due to prion infection or advanced age.

In summary, the detection of PrPSc by both immunoblot and IHC in 4 of 8 mice studied between 593 and 762 dpi supported the conclusion that transmission of prion disease occurred in this experiment using brain from the Y226X patient. The finding that only half the mice were positive, even after such a long observation period, suggested that the amount of infectivity transferred was near the end-point of the sensitivity of this transmission system.

### Transmission experiments using brain tissue from a patient expressing a Q227X PrP mutation.

Transmission was also attempted using homogenate made from frozen brain tissue from a patient with PRNP mutation, Q227X. This mutation resulted in expression of truncated PrP with one additional amino acid residue compared to the PrP expressed by the Y226X patient. The Q227X patient presented at age 42 with a hypokinetic rigid syndrome, and was diagnosed with frontotemporal dementia [23]. The patient subsequently developed tremors and seizures and mutism, and died 6 years after onset of disease. Neuropathology showed numerous multicentric and unicentric amyloid plaques which stained positive with anti-PrP antibodies. Plaques were not associated with blood vessels, but were mostly in the neuropil in numerous brain regions.

**Fig. 3:**
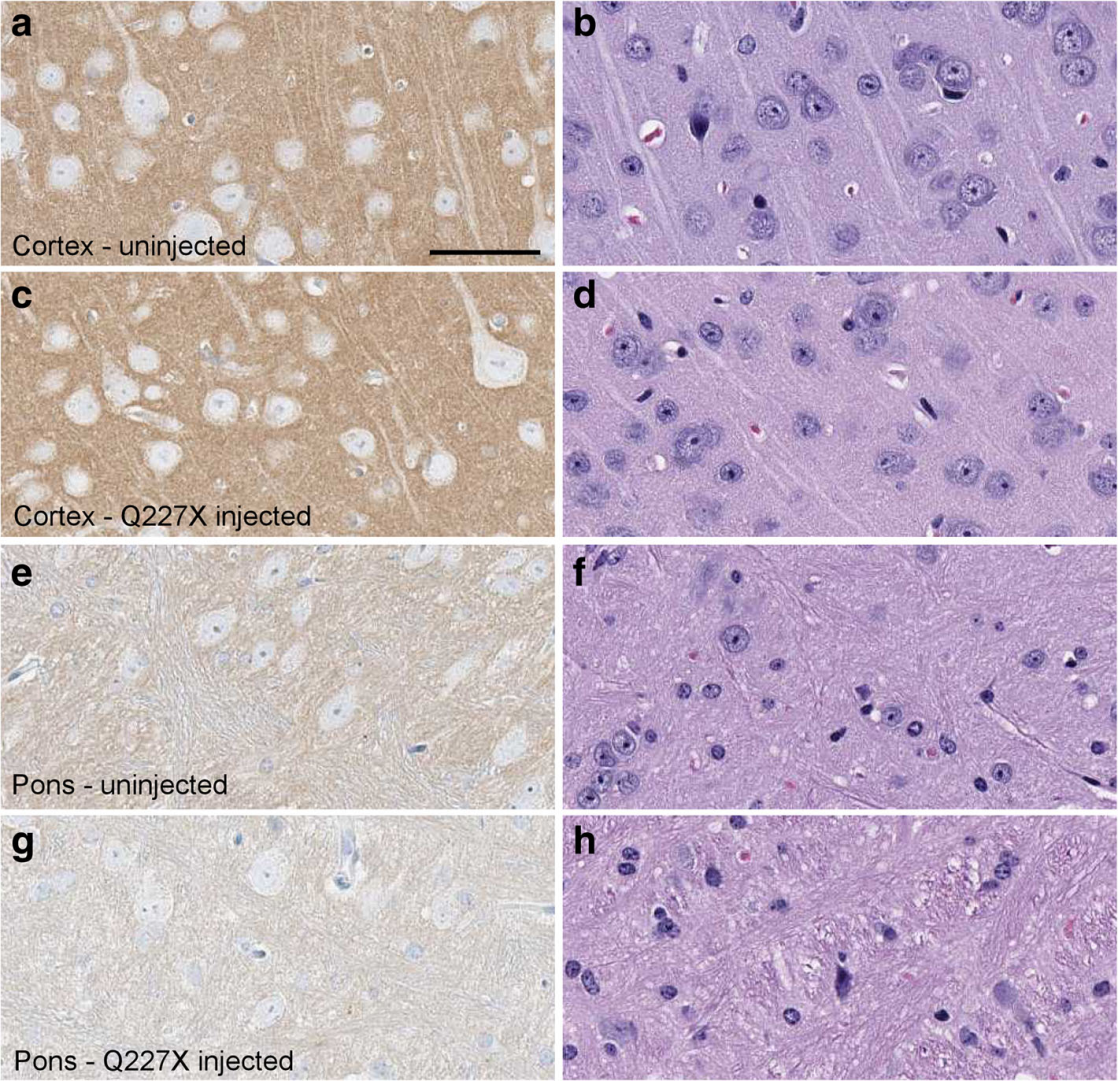
Histology and immunohistochemical staining of PrP in two brain regions of a tg66 mouse injected with Q227X brain homogenate at 743 dpi, and an uninfected aged control tg66 mouse (age 649 days). Panels **a**, **c**, **e**, **g** show PrP staining with biotinylated-3F4 antibody, and panels **b**, **d**, **f**, **h** show H&E staining. Uninfected cortex (**a**, **b**), Q227X injected cortex (**c**, **d**), uninfected pons (**e**, **f**), Q227X-injected pons (**g**, **h**). Panels **a** and **c** show darker tan staining than panels **e** and **g** due to a higher level of background PrPC in cortex compared to pons. No prion disease vacuoles or significant deposits suggestive of PrPSc were observed in uninfected or the Q227X-injected mice. Scale bar in panel **a** is 50 μm and is valid for all panels

Following intracerebral injection of tg66 mice with brain homogenate of this patient, mice were followed for clinical status weekly, but no mice with clinical signs suggestive of prion disease were noted at any time (Table 3). Individual mice were euthanized at various time-points between 77 and 798 dpi, but no PrPSc was detected in any mice by immunoblotting of brain tissue (Fig. 1b). IHC examination of brain for PrPSc deposition was also negative, and there was no gray matter vacuolation typical of prion diseases (Fig. 3). Thus, there was no evidence for transmission of prion infectivity to tg66 mice from the brain tissue of the Q227X patient.

**Table 3:** Transmission study of human, genetic mutant Q227X PrP to tg66 transgenic mice expressing human PrP

Mouse number	DPI	PrPSc IHC	PrPSc western blot	Clinical TSE suspect	Clinical notes (reason for euth) & relevant necropsy findings
B302–2	77^a^	–	nt	No	normal
B299–1	77^a^	–	nt	No	normal
B302–3	239^a^	–	nt	No	normal
B302–4	239^a^	–	nt	No	normal
B304–1	529^a^	–	nt	No	normal
B304–2	529^a^	–	nt	No	normal
B630–1	545	–	–	No	lung neoplasia
B296–1	650	–	–	No	eye neoplasia
B295–1	696	–	–	No	injured, thin, ataxic, adequate nesting, lymphoma
B296–2	716	–	–	No	injured, thin, barrel rolled twice, aware and responsive
B295–2	743	–	–	No	distended abdomen, liver neoplasia
B296–3	756	–	–	No	thin
B306–3	782^a^	–	nt	No	normal
B306–4	782^a^	–	nt	No	normal
B340–1	784	–	–	No	urine scalding
B295–3	798	–	–	No	normal

^a^- Indicates mice that were stereotactically microinjected into the striatum. Mice injected using this technique were euthanized electively and tissues were processed to directly screen the injection needle track and adjacent brain by IHC for any PrP replication. Mice without asterisks were intracerebrally inoculated with a 30ul volume of brain homogenate and euthanized when they developed neurologic signs consistent with prion infection (none) or conditions requiring euthanasia for humane reasons or at the termination of the experiment at 782 and 798 dpi

### Transmission experiments using brain tissue from a patient expressing a G131V PrP mutation

A third patient with a rare PRNP mutation, G131V, was also studied for transmission [22]. In this patient, the disease onset was at age 36 and the patient died after 15 years of progressive clinical disease including personality changes, memory impairment, loss of cognition, tremor, ataxia, myoclonus, and bradykinesia. Neuropathology showed numerous deposits of PrP amyloid in gray matter of cerebrum, cerebellum and midbrain without evidence for spongiform degeneration. Another patient with the same mutation was reported previously [30].

**Fig. 4:**
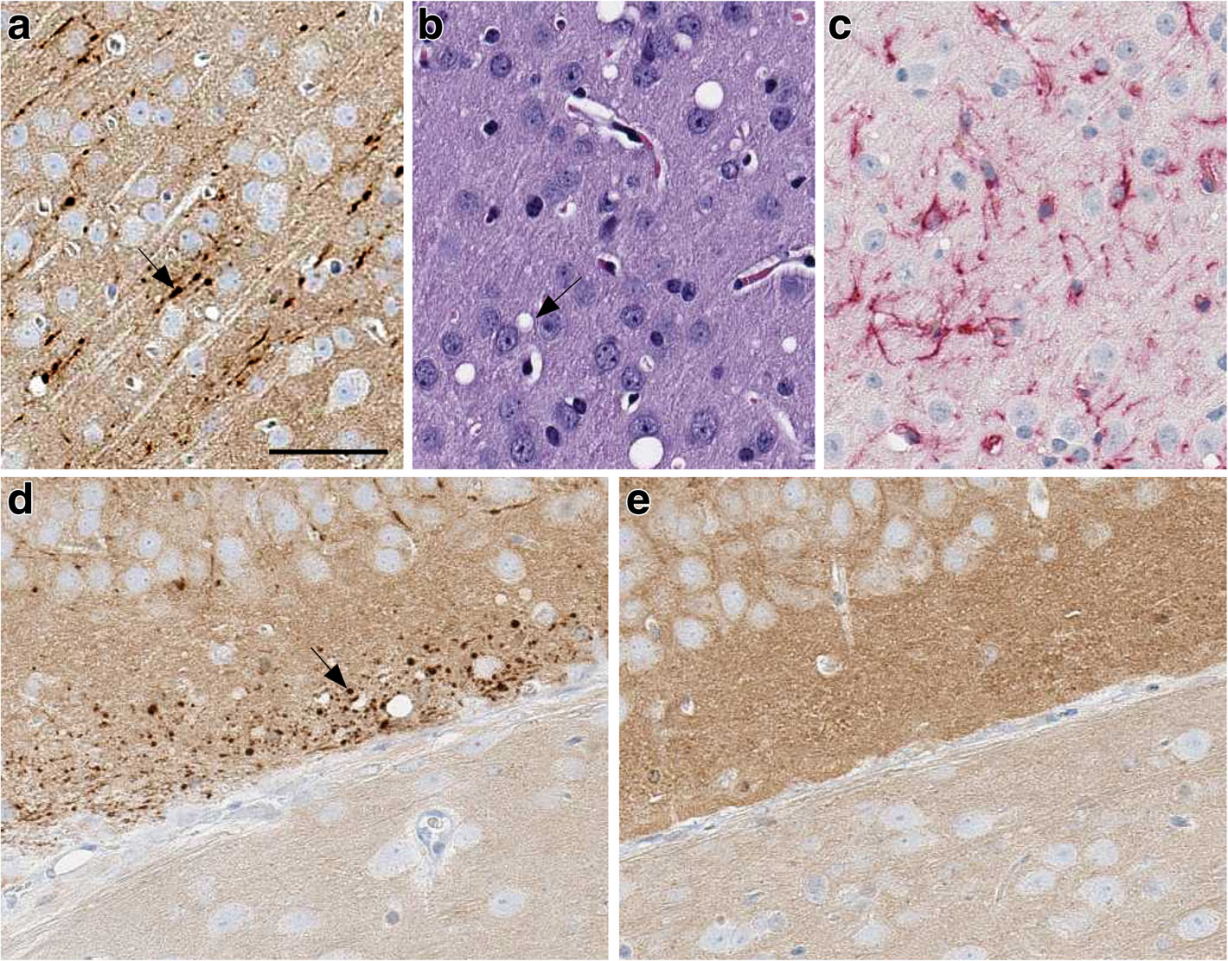
Immunohistochemistry and neuropathology of G131V-injected tg66 mice at 531 days post injection. In cerebral cortex: Linear axonal staining of PrPSc (arrow) was detected by antibody 3F4 (**a**). Vacuoles (arrow) shown by H&E staining (**b**) and microglia seen with anti-Iba1 staining (**c**) were also detected. In the Oriens layer of hippocampus of the same mouse shown in panels **a**, **b**, **c**, punctate 3F4 staining of PrPSc was detected in a pattern possibly indicating association with axonal cross-sections in this region (arrow) (**d**). As a negative control, in the same region of a mouse injected with Q227X brain homogenate no PrPSc deposits were observed at 529 dpi (**e**). The scale bar shown in panel **a** is 50 μm and applies to panels **a**-**e**

**Fig. 5:**
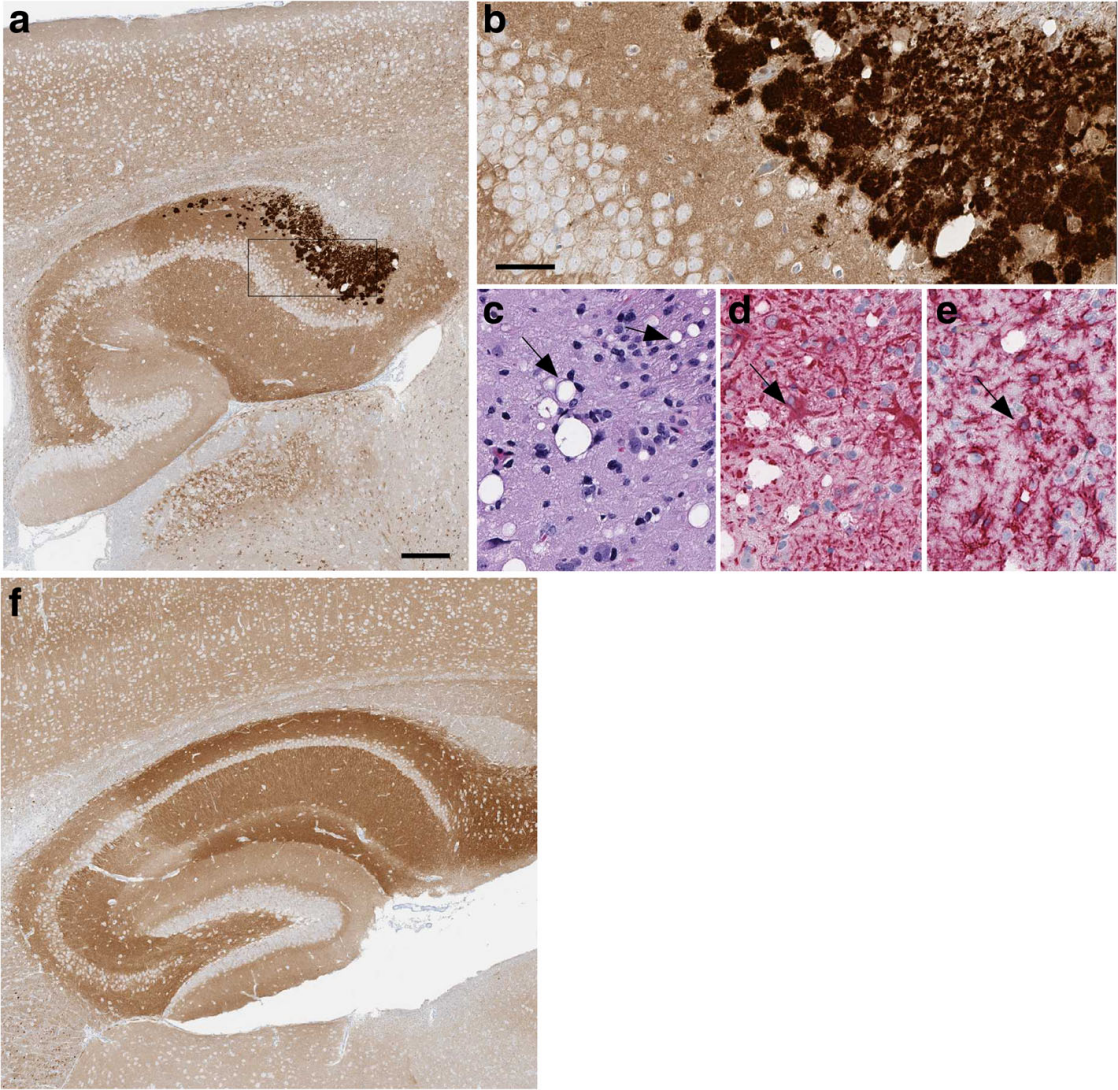
Immunohistochemistry and neuropathology of a G131V-injected tg66 mouse at 731 days post-injection. **a**-**e** Oriens layer of dorsal hippocampus. Coarse plaque-like staining of PrPSc by antibody 3F4 is seen at low (**a**) and high (**b**) magnification. Rectangular box in a denotes area seen in b. In the same area, gray matter vacuoles (arrow) were noted by H&E staining (**c**), and astrogliosis (**d**) and microgliosis (**e**) were observed (arrows) by IHC using antibodies to GFAP and Iba1. Panel **f**: No PrPSc deposits were seen in a control age-matched uninfected mouse in the same hippocampal region shown in (**a**) The scale bar in panel **a** is 200 μm and applies to panel **f**. In b the bar is 50 μm and applies to panels **b**-**e**

For transmission experiments, homogenate from frozen brain was injected into tg66 mice, and mice were followed as described above. By IHC PrPSc was detected in brain of 11 of 11 mice tested between 531 and 784 dpi (Table 4). At earlier time-points (two mice at 531dpi) PrPSc deposits appeared as linear axonal staining in the cerebral cortex (Fig. 4a), and gray matter vacuoles and microglia were noted in the same areas (Fig. 4b and c). In these same mice, PrPSc deposits were detected as small round objects consistent with axonal cross-sections in the Oriens layer of the ventral HC (Fig. 4d). At later time-points, such as 731 dpi, the deposits in the cerebral cortex had the same fine linear axonal pattern as at 531 dpi (not shown). However, at 731 dpi, PrPSc deposits in the Oriens layer of the dorsal hippocampus had a bulky coarse pattern (Fig. 5a and b). In these PrPSc-positive areas, vacuolation of neuropil (Fig. 5c) and astro- and micro-gliosis were observed (Fig. 5d and e).

**Table 4:** Transmission study of human, genetic mutant G131V PrP to tg66 transgenic mice expressing human PrP

Mouse number	DPI	PrPSc IHC	PrPSc western blot	Clinical TSE suspect	Clinical notes (reason for euth) & relevant necropsy findings
B300–3	79^a^	–	nt	No	normal
B339–3	79^a^	–	nt	No	normal
B307–1	241^a^	–	nt	No	normal
B307–2	241^a^	–	nt	No	normal
B308–3	531^a^	+	nt	No	normal
B328–1	531^a^	+	nt	No	normal
B633–1	596	+	–	No	normal
B297–1	650	+	–	No	weight loss, hind limb weakness
B297–2	731	+	–	Yes	head bob, tremors, thin
B298–1	731	+	–	Yes	weak hind legs, thin, kyphosis, weepy eye, ataxia for 1 month
B297–3	735	+	–	Yes	thin, slight wobble, rough coat, squinty eyes, decent nest
B297–4	735	+	–	Yes	thin, slight wobble, rough coat, squinty eyes, decent nest
B632–1	758	+	–	Yes	thin, rough coat, mild ataxia
B298–2	769	+	–	Yes	thin, ataxic
B303–1	784	+	–	Yes	urine scalding, weak hind legs, tremor

^a^- Indicates mice that were stereotactically microinjected into the striatum. Mice injected using this technique were euthanized electively and tissues were processed to directly screen the injection needle track and adjacent brain by IHC for any PrP replication. Mice without asterisks were intracerebrally inoculated with a 30ul volume of brain homogenate and euthanized when they developed neurologic signs consistent with prion infection or conditions requiring euthanasia for humane reasons

In contrast to PrPSc detection by IHC, PrPSc was not noted in immunoblotting experiments in any of the mice tested, even when the ultrasensitive PTA precipitation method was used (Fig. 1b). However, clinical signs suggestive of prion disease were observed in 7 of the 11 mice which scored positive for PrPSc by IHC (Table 4). Tremors, ataxia, wobbly gait hind leg weakness and kyphosis were among the common signs noted. Nevertheless, due to the advanced age of these mice, we could not exclude the possibility that some or all of these signs were related to old age rather than prion infection.

Despite the lack of PrPSc detection by immunoblot and the difficulty with interpretation of clinical signs, we feel that the PrPSc detection by IHC associated with axons and hippocampal aggregates was suggestive of prion transmission to tg66 mice, but final interpretation will require the completion of the second passage experiments.

### RT-QuIC analysis of brain tissue derived from first passage tg66 mice inoculated with Y226X, Q227X and G131V human brain tissue

**Fig. 6:**
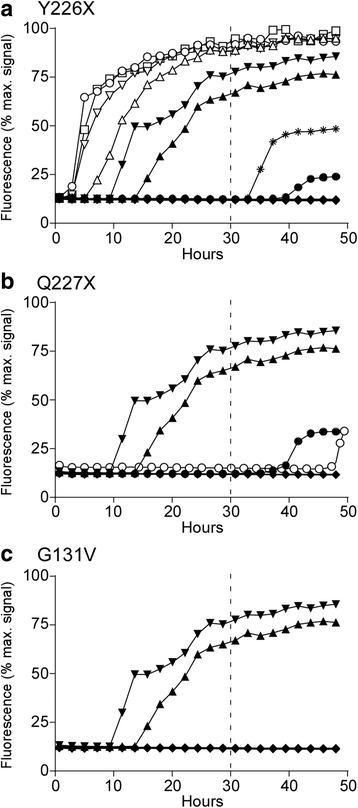
RT-QuIC analysis of tg66 mouse brain following inoculation with human brain expressing GSS mutations Y226X, Q227X and G131V. RT-QuIC was performed on tg66 mouse brain homogenates using bank vole recPrP substrate. Curves show the average of the fluorescence values for 4 replicate wells at each time-point. In each panel, two tg66 mice inoculated with vCJD were run as positive controls (▲ and ▼). **a** Y226X-inoculated tg66 mice. Open symbols show the four Y226X inoculated tg66 mice which were PrPSc-positive by immunoblot and IHC (B322–1, B323–1, B323–3, B324–1). The asterisk and black circle depict two Y226X mice (B322–2, B324–2) which gave low level fluorescence in one well out of 4 after 30 h and did not meet criteria for a positive reaction (see Methods). Two other Y226X mice (B323–2, B326–5), which were negative for PrPSc by immunoblot and IHC, and two uninoculated tg66 mice are collectively shown with the solid black diamond symbol, and all four had no fluorescence above background. **b** Q227X-inoculated tg66 mice. Two mice (B295–2, B296–2) had low level fluorescence between 40 and 50 h but did not meet criteria for a positive reaction. The remaining four Q227X mice tested (B295–1, B295–3, B296–3, B340–1) and two uninoculated tg66 mice were negative and are shown with the solid black diamond. **c** G131V-inoculated tg66 mice. The seven G131V-inoculated tg66 mice tested (B297–2, B297–3, B297–4, B298–1, B298–2, B303–1, B632–1) and two uninoculated tg66 mice all showed no seeding activity, indicated by the solid black diamond

To screen for PrP amyloid seeding activity in tg66 brains we performed RT-QuIC analysis on brain homogenates from tg66 mice inoculated with each GSS mutant. For the RT-QuIC assay we used bank vole as a substrate and followed established protocols [29]. Brains derived from 2 tg66 mice infected with vCJD were run as positive controls (Fig. 6). Brain tissue from the 4 Y226X-inoculated mice that previously had detectable PrPres deposition showed strong seeding activity following a short lag phase (less than 30 h) in 4/4 wells tested for each mouse (Fig. 6). In contrast, brains from Y226X-inoculated mice that tested negative for prion deposition by WB and IHC did not show significant seeding activity by RT-QuIC prior to 45 h of testing. Interestingly, despite the presence of PrPres deposition by IHC, none of the 7 G131V-inoculated tg66 mice tested by RT-QuIC had detectable seeding activity. Six Q227X-inoculated tg66 mice were also screened by RT-QuIC and did not have detectable seeding activity. Thus, transmission of the G131V and Q227X diseases to tg66 mice was not supported by these RT-QuIC data (Fig. 6).

### Second passage transmission experiments using brain tissue derived from tg66 mice inoculated with Y226X, Q227X and G131V

To test for subclinical disease in Q227X-inoculated tg66 mice and to confirm the transmission of the Y226X and G131V mutants to tg66 mice, we performed a second passage into tg66 mice. For each original mutant, brain homogenates from three first pass tg66 mice were intracerebrally inoculated into groups of 9–12 recipient tg66 mice. To date, one donor mouse (Y226X #B322–1) has caused clear clinical prion disease in 100% of challenged mice with an incubation period range of 167–185 dpi. Compared to the first passage, this incubation period is reduced by over 400 days, suggesting a rapid adaptation to tg66 mice and/or that the initial tg66 mice were inoculated with a very low titer. The other groups of experimental mice range from 40 to 225 days post-inoculation. Given the extended incubation periods observed on first passage final conclusions cannot be drawn at this time, and may require an additional two years of observation.

## Discussion

In the present experiments, we observed transmission of 1 new familial prion disease-associated mutation, Y226X, after injection of human patient brain tissue into tg66 transgenic mice overexpressing human PrP. Transmission was indicated by detection of PrPSc deposits, typical prion spongiform degeneration and gliosis in brains of recipient mice, detection of PrPSc by immunoblot and PrP amyloid seeding activity by RT-QuIC assay in 4 of 8 tg66 recipients. To confirm these data a second passage in tg66 mice has been started and one first passage Y226X tg66 brain which was positive in the other tests has already caused clinical disease in 9/9 mice in the second pass from 167 to 185 dpi, thus confirming the first passage transmission results. Transmission from Y226X patient brain is the first example of transmission from a human patient with a mutation involving truncation of PrP. No transmission was observed in previous attempts using Y145X or Y163X brain tissues [27, 44]. and in the present work, we also saw no transmission using brain from a Q227X patient.

In the current study, transmission of disease by inoculation of G131V human brain was also suspected as 11 of 11 tg66 recipient mice showed suggestive clinical signs and convincing PrPSc deposits in brain tissue between 531 and 784 dpi. However, these brains were not positive for PrPSc by immunoblot and did not show PrP amyloid seeding activity by RT-QuIC assay. One additional mutation Q227X, was also tested in the present study and was negative by all parameters measured so far in first passage tg66 mice. Second passages of tg66 mice inoculated with both the G131V and Q227X brain tissue are in early stages, and no positive conclusions can be drawn at this time.

Present and previous data together suggest that not all patients with familial prion disease have infectious prions. Possibly certain PrP mutations do not form a conformation capable of generating an infectious moiety. Alternatively, there might be a low titer of infectivity in the tissues which have not shown transmission. So far there has been no side by side comparison of transmission of familial prion disease brain infectivity using the sensitive bank vole animal model [34] or various human PrP expressing mice presently available. Regardless of which system is used, negative transmission results can always be interpreted as being due to an inefficient recipient animal system. However, in the present experiments using tg66 mice, if infectivity is present in Q227X patient brain, the titer must be below the level in the Y226X patient.

The sensitivity of tg66 mice to transmission of human sCJD and vCJD was reported previously [37, 38], and this sensitivity was emphasized in the present study by the transmission of infectivity from Y226X brain obtained from formalin-fixed tissue sections which were removed from microscope slides. Previous experiments have shown that prion infectivity is only partially sensitive to treatment with formalin [40], and can be obtained from formalin-fixed brain [2, 15, 32, 35]. In the case of Y226X, formalin-fixed brain was the only tissue available, even in the initial description of this patient [23]. In our experiments with Y226X brain, positive transmission was seen in only four of the eight mice observed for > 590 days. Thus, there was approximately 1 ID50 in the 30 μl volume of 1% brain homogenate injected into these mice. This titer appeared to be at the threshold of our ability to detect transmission in the tg66 mouse system.

In the present work two different injection methods were used for transmission. For standard macroinjection, 30 μl of 1% brain homogenate was manually injected in the parietal region, and for microinjection, 1 μl of 10% brain homogenate was injected stereotactically into the striatum. Previously, we used the microinjection system to detect PrPSc in striatum as early as 30 min post-injection [9]. Therefore, we reasoned that using the needle track wound as a guide in this transmission system, we might find PrPSc near the needle track at very early times and thus shorten the experiment. Unfortunately, this did not turn out to be the case, and even in the few microinjected mice which were positive (531dpi mice injected with G131V brain) the PrPSc was detected in the cortex and hippocampus rather than the striatum (Figs. 4 and 5). Therefore, the biological preference for another brain region appeared to overcome the fact that the injection was placed at a specific location in the striatum.

PrPSc deposits in mice injected with Y226X brain were found mainly in pons, thalamus and midbrain. Deposits varied in character from large plaque-like deposits to coarse or medium deposits in perineuronal sites or neuropil (Fig. 2 and Table 2). In different mice deposits in the same region, i.e. pons, were sometimes different (Fig. 2a versus 2b). Interestingly, no mice had perivascular PrPSc which were very common sites in the original human patient. Thioflavin S staining for amyloid showed that some, but not all, of the large plaque-like deposits in tg66 mice were positive (Fig. 2, panel a-2, insert). This was markedly different from the pattern of perivascular amyloid and CAA seen previously in scrapie-infected tg44 mice expressing truncated anchorless PrP [8, 36, 39]. Thus, the formation of abundant PrPSc amyloid in the Y226X patient appeared to be dependent on the synthesis of anchorless PrP in this patient. In contrast, amyloid PrPSc was minimal in the tg66 mice in the present transmission experiments apparently due to the low amount of anchorless PrP made by these recipient mice.

In the mice injected with G131V brain, PrPSc deposition, spongiform degeneration and gliosis were observed in all eleven mice examined at timepoints starting at 531dpi. PrPSc was found mainly in the cerebral cortex and hippocampus. At 531 dpi, PrPSc appeared to be associated with axons giving a linear pattern in the cerebral cortex and a punctate pattern in the Oriens layer of the hippocampus (Fig. 4d-f). This difference in morphology appeared to depend on whether the plane of section was across or parallel to the axons involved. The linear pattern in cortex was similar to the morphology observed in the original patient. At 731dpi, the fine linear PrPSc pattern was still seen in cerebral cortex, but in the Oriens layer of the hippocampus, there were both large plaque-like deposits and smaller punctate deposits, which suggested more extensive axonal dystrophy and possibly extra-axonal plaque-like deposition. Despite the plaque-like size of some of these deposits, they did not stain with Thioflavin S suggesting they were not amyloid.

Two patients with the G131V mutation have been published previously [22, 30]. In our transmission studies, which used tissue from the second patient, all the tg66 mice injected with G131V patient brain were negative for PK-resistant PrPSc by immunoblotting and PrP amyloid seeding activity by RT-QuIC assay, but all had detectable PrPSc by IHC. There reasons for these discrepancies are not known, but the lack of a confirming biochemical test for PrPSc or seeding activity, weakens the interpretation that transmission was truly positive. Perhaps the second passage in tg66 mice will be able to clarify these conclusions.

The transmissible agent detected in human patients with Y226X PrP and possibly also with G131V PrP is likely to contain aggregates of the mutant PrP expressed in these patients. However, both these patients, similar to most known familial prion disease patients, are heterozygous for the mutant PrP allele, and thus they express both normal and mutant PrP. Presence of the non-mutant PrP isoform has been associated with insoluble aggregates of mutant PrP in some patients with familial prion diseases [7, 14, 41, 46]. Thus, it is possible that the normal and mutant PrP isoforms present in familial prion disease patients may both contribute to the disease pathogenesis and/or generation of a transmissible agent. Similarly, we have previously described the influence of co-expressed normal and mutant PrP alleles (expressing anchorless PrP) in scrapie-infected transgenic mice resulting in deposits of both nonamyloid and amyloid PrPSc as well as more rapid disease progression [10].

In summary, the present results bring to 13 the number of PrP point mutations associated with familial prion disease which have been tested for transmission. Of these, 9 mutations have showed evidence for transmission to primates or rodents. PrP Y226X is the first PrP mutation encoding a truncated PrP molecule which was transmissible. In contrast, the other truncated PrP mutant tested here, Q227X, showed no evidence for transmission which was similar to two other truncation mutants previously tested by others. These differences in transmission might be due to the influence of specific protein folding structural factors on generation of infectivity, or alternatively, they might be due to biological statistical variation in the quantity of prion infectivity present in the brain samples tested.
